# Reusability of Germany´s total diet study food list upon availability of new food consumption data—comparison of three update strategies

**DOI:** 10.1038/s41370-023-00522-4

**Published:** 2023-02-01

**Authors:** Anna Elena Kolbaum, Sebastian Ptok, Christian Jung, Lars Libuda, Oliver Lindtner

**Affiliations:** 1 German Federal Institute for Risk Assessment (BfR), Max-Dohrn-Straße 8-10, 10589 Berlin, Germany; 2 Paderborn University, Faculty of Natural Sciences, Institute of Nutrition, Consumption and Health, Warburger Straße 100, 33098 Paderborn, Germany

**Keywords:** BfR MEAL Study, Food monitoring, Food sampling, Food consumption, KiESEL study, EsKiMo II study

## Abstract

**Background:**

The German total diet study (TDS)—BfR MEAL Study—established its food list in 2016 based on food consumption data of children (0.5–<5 years) and adults (14–80 years). The list consists of 356 foods selected for analysis in order to ensure ≥90% coverage of the diet. Recently, new food consumption data for children (0.5–<6 and 6–<12 years) in Germany became available, which raised the opportunity to evaluate the applicability of the MEAL food list 2016 on new data.

**Objective:**

We tested the hypotheses that the MEAL food list 2016 also covers ≥90% of the diet of the new collected food consumption data, and that the selection of foods from younger children and adults was sufficient to also cover the middle age group (6–<12 years). Strategies for updating the existing food list were assessed.

**Methods:**

Three approaches evaluated the reusability and potential adjustment strategies of the existing food list. Approach 1 applied the existing food list to new food consumption data. Approach 2 allowed the extension of the existing food list to improve coverage of food consumption. Approach 3 set up a new food list based on the new data.

**Results:**

The MEAL food list 2016 covered 94% of the overall diet of the new collected food consumption data. The diet of the middle age group was sufficiently covered with 91%. However, coverage on main food group or population subgroup level was <90% in some cases. Approach 3 most accurately identified relevant modifications to the existing food list. 94% of the MEAL food list 2016 could be re-used and 51 new foods were identified as potentially relevant.

**Significance:**

The results suggest that a high investment in the coverage of a TDS food list will lower the effort and the resources to keep data updated in the long-term.

**Impact:**

There is no established approach to update a TDS food list. This study provides comparative approaches to handle newly collected food consumption data for follow-on TDS activities. The results provide useful information for institutions planning or updating a TDS. Furthermore, new food consumption data for children in Germany recently became available and are here presented for the first time.

## Introduction

Total diet studies (TDS) aim to assure that substances in foods are at safe levels at long-term exposure. They are the recommended tool by EFSA, WHO and FAO to assess the background contamination in nearly the entire diet of a population of interest [1]. Only the assessment of the total diet allows useful risk characterization because the comparison with the respective toxicological reference values is then based on the overall dietary exposure. Consideration of the total diet further allows identification of contribution from each measured food, which is especially important when discussing risk management measures for specific foods or food groups. A TDS consists of three basic characteristics: covering at minimum 90% of the total food intake (g/kg body weight) of the population of interest, preparing foods ‘as consumed’ and pooling similar foods to composite samples prior analysis [1]. The TDS food list is the core of the study. The selection of the foods and the aggregation level will determine the level of detail of the exposure assessment. Nevertheless, the extent of the food list is always a trade-off between scientific needs and resources. The core food list of the German TDS, the BfR MEAL Study (‘meals for exposure assessment and analysis of foods’), was established in 2016 and includes 356 foods [2, 3]. Much effort and resources were put into a stepwise approach of aggregating and disaggregating foods from different food consumption surveys under consideration of the occurrence and variability of about 300 substances. As many other TDS, the BfR MEAL Study was initiated as a singular project. Therefore, the aim was that the food list should be able to cover the food consumption also in the long-term with sufficient detail. In addition, updates of food consumption data are often collected at irregular intervals and a well-developed food list is assumed to compensate potential changes in consumption habits until new data become available. For example, the currently used food consumption data for adults in Germany are from 2005/2006, which replaced data collected 20 years earlier [4], and until recently, the 2001/2002 collected food consumption data from the VELS study were the most recent data for children [5]. Both datasets have been used to compile the MEAL food list in 2016. Egan et al. (2007) [6] highlighted the necessity to consider changed consumption habits in a TDS by a comparison of exposure estimates based on the 1990 and 2003 US FDA’s food lists. Both the changed food consumption amounts but also the accordingly adapted food list revealed an up to 46% increase of cadmium exposure in the US population.

With the KiESEL study, new food consumption data for children (0.5–<6 years) became available for Germany [7]. Based on the abovementioned statements, the hypothesis is that the MEAL food list 2016 also covers >90% of more recent food consumption data.

In the meanwhile, also new food consumption data for adolescents (6–<12 years) became available from the EsKiMo II study [8]. This age group has not been considered for the food list yet, due to the assumption that food consumption in relation to body weight will range between that of young children and adults. The second hypothesis therefore is that by considering young children and adults for the compilation of the MEAL food list 2016 the food consumption of the middle age group (EsKiMo II study) was also sufficiently covered.

This work tested the above-mentioned hypotheses and evaluated in three different approaches strategies to improve the food list when including new data. The results can advise future TDS activities in Germany or other countries for an efficient use of resources when developing or adjusting a food list.

## Materials and methods

### Food consumption data

Food consumption data used to establish the MEAL food list 2016 were the VELS study for children aged 0.5–<5 years and data from the National Nutrition Survey II (NVS II) for adults aged 14–80 years. Details on methods and data collection are described in Banasiak et al. (2005) [5] and Krems et al. (2006) [9], respectively. In brief, the VELS survey monitored food consumption of 804 individuals during 2001/2002. Data were collected by completing two-times a 3-day weighted dietary record. The NVS II was conducted 2005/2006. For compiling the food list, data from 13,926 participants who completed 24-h recalls on two non-consecutive days were used.

For the here presented re-evaluation and further development of the MEAL food list 2016, recent data from the KiESEL study (Children’s Nutrition Survey to Record Food Consumption) for children aged 0.5–<6 years and the EsKiMo II study (Eating study as a KiGGS Module in KiGGS Wave 2) for children aged 6–<12 years were used. The KiESEL study included 1008 subjects whose legal guardians completed weighted dietary records on three days plus one non-consecutive day in 2014–2017. The KiESEL study involved 56 individuals that were at least partially breastfed. Since no information regarding the volume of the human milk was available, the present evaluation considered only data from non-breastfed children (*N* = 952). From the EsKiMo II study, data from 1190 children aged 6–<12 years were used who completed a three plus one day weighted dietary record. Data were collected 2015–2017. Details are described in Nowak et al. (2022) [7] and Brettschneider et al. (2018) [8].

All surveys provided sociodemographic and anthropometric data on individual basis.

### MEAL food list 2016

To establish the MEAL food list 2016 data for children (VELS) and adults (NVS II) were assessed separately. In a first step, related components were aggregated to foods as consumed (e.g., instant tea plus water) to correctly assess mostly consumed foods. One principle in establishing the food list was to avoid the assignment of foods to a main food group if they contained components of two or more main food groups. Therefore, composite dishes containing separate prepared components from different main food groups, like pasta with tomato sauce, where broken down into their components. The high number of food items used in food consumption data were reduced by defining a grouping within each of the 19 FoodEx2 main food groups^1^ (e.g., ‘apple’, ‘banana’ or ‘strawberries’ in the main food group ‘fruit and fruit products’). Attributes like food species, kind of preparation, packaging, ingredients as well as expected substance levels and variability were considered in the process of food grouping (e.g., ‘grapes’ and ‘raisins’ were kept separately due to differences in water contents).

For each food, the consumption per kg body weight was determined on individual basis and subsequently averaged for different age groups (children) or age/sex groups (adults) separately. For selection which of the grouped foods are relevant for the MEAL food list 2016, foods were sorted in descending order related to their consumption within each main food group and for each age/sex group. For each of the age/sex groups the lowest number of foods was selected which cumulatively covered ≥90% of respective mean intake per main food group (equal to foods with highest mean intake). Further foods were added to the temporary food list, if they were focused in German risk assessments in the years before (e.g., ‘sheep liver’). To optimize the food list and to reduce the final number of foods, foods present in only few age/sex groups and with low mean consumption were either excluded or aggregated with similar foods. With respect to the consideration of food trends, which were not relevant at the date of the food consumption studies, further foods like ‘pseudo cereals’ were also included in the food list. After the selection process, the final MEAL food list 2016 consisted of 356 foods (called ‘MEAL foods’ in the following; Supplementary Table S1).

#### Comparison of food consumption data

The first attempt was to uncover the changes in food consumption reported 2001/2002 (VELS) and 2014–2017 (KiESEL). Only non-breastfed children were selected for evaluation. While the whole KiESEL study population of 0.5–<6 years was applied for the food list assessment, the population was restricted to <5 years for the comparison with the VELS age groups. Mean food consumption (grams per kilogram body weight (bw) and day; g/kg bw/day) was estimated by averaging over reporting days on individual basis with subsequent averaging over main food groups per age group. Mean food consumption is reported for consumers only. For KiESEL weighting factors correcting for age, sex, region, regional structure, distribution of weekdays and parental education were applied, in order to ensure representativeness for children in Germany [7]. No weighting factors were available for the VELS study.

### Approaches for updating the MEAL food list 2016

**Fig. 1:**
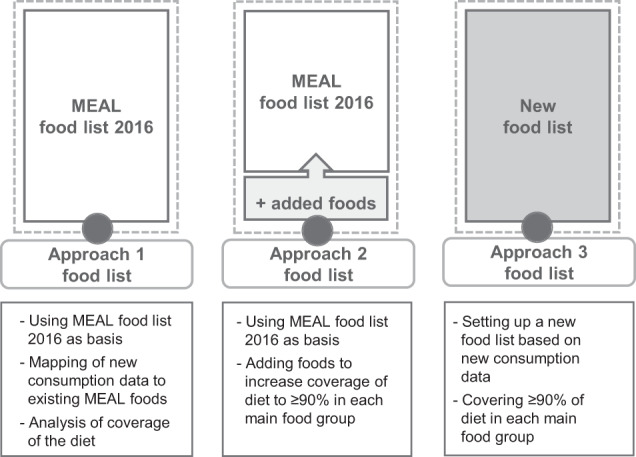
Update strategies. Outline of the three approaches tested in order to update the MEAL food list 2016.

The abovementioned hypotheses were tested in three approaches (Fig. 1).

In *approach 1*, the existing MEAL food list 2016 was strictly followed for aggregating the consumed foods of the new surveys. Food records from the KiESEL and the EsKiMo II study were assigned to the 356 MEAL foods according to the method as reported for compiling the MEAL food list 2016. No new MEAL foods were established. In addition, the total food consumption per age group and per main food group was assessed in order to estimate the proportion (%) of covered consumption. This approach showed whether the existing food list complies with the TDS principle to cover in total at least 90% of food consumption for newly collected consumption data (KiESEL) (hypotheses 1). It also revealed if this also applies on a more detailed level, assessing the coverage per age group and per main food group. Furthermore, approach 1 tested whether this was also achieved for the middle age group (EsKiMo II) (hypotheses 2).

*Approach 2* used the existing food list as base, but allowed for extension for further foods. Where coverage of the ‘Approach 1 food list’ was <90% in a main food group or age group, further foods were added according to their consumption amount until 90% was achieved. To do so, the remaining foods, that could not be assigned to one of the existing 356 MEAL foods, were aggregated to additional MEAL foods. The same aggregation scheme as for the MEAL food list 2016 was followed. In addition, foods with low consumer rate (<5%) were removed, as long as 90% consumption per main food group were still covered for each age group. This aimed to narrow the food list while at the same time meeting the 90% criterion. Consumer rate was defined as percentage of individuals with consumption on the total number of individuals in the same age group. For this approach, the 5% consumer rate cut-off criterion was just applied for foods not contained in the food list yet, in order to keep the structure of the initial food list. This procedure allowed foods with <5% consumers to be included in the foods list. In order to compare and discuss the impact of these low-consumer foods, a further evaluation excluding all foods with <5% consumers is displayed in the Supplementary Material and discussed along with the results. Approach 2 showed what measures are necessary to improve the coverage on a more detailed level.

In *approach 3*, a new food list was compiled independently from the existing food list. For each main food group the foods were selected according to their consumption amounts until 90% for each age group was achieved. Additional foods potentially relevant for exposure were included also if consumption amount or frequency was low. To identify these foods, risk assessments performed by the BfR since compilation of the food list 2016 were reviewed. Since this food list only considered foods relevant for children and adolescents (KiESEL and EsKiMo II), the selected foods were finally supplemented with those foods derived for the adults (14–80 years; NVSII) based on the initial food list. Again all foods with <5% consumers were removed or further aggregated, as long as 90% per age group were still covered. This approach considered changes in food consumption pattern. Foods no longer relevant were identified and removed from the food list. Others were added as representative part of the diet.

### Data analysis

To compare the food consumption data reported in 2001/2002 (VELS) and 2014–2017 (KiESEL) a weighted Mann–Whitney *U* test with a significance level of *p* < 0.05 was used.

The statistical software R version 4.1.1 (R Foundation for Statistical Computing, Vienna, Austria) and Microsoft Office Excel 2016 (Microsoft Corporation, Redmond, WA, USA) were used for all calculations.

## Results

### Comparison of food consumption data

Considering the total population of the VELS survey 2005/2006 and the KiESEL survey 2014–2016, the total food consumption reported in the KiESEL study was 6% higher compared to the VELS study (Table 1). Greatest increase was observed in the main food group ‘Products for non-standard diets, food imitates’ (e.g., soy drink, vegetarian sausages) (+45%). Highest decrease was observed for ‘Food products for infants and toddlers’ (−35%). In terms of age groups, 3–<5 year old children showed highest increase in total food consumption (+10%), followed by the age groups 1–<3 and 0.5–<1 years, with 7 and 5% increase, respectively. However, the latter was not statistically significant. Notably, in all age groups, the relative consumption of ‘Products for non-standard diets, food imitates’ showed greatest change. It decreased by −677% for 0.5–<1 year old children (not significant), but significantly increased with age to a plus of 58% for 3–<5 year old children. Some notable changes were also a significant increase in the consumption of ‘Water and water-based beverages’ or ‘Eggs and egg products’, as well as a decline in ‘Milk and dairy products’.

**Table 1:** Comparison of mean food consumption (g/kg bw/day; consumers only) between children surveyed 2001/2002 (VELS study) and 2014–2017 (KiESEL study).

Age group	Total; 0.5–<5 y.					0.5–<1 y.				
Survey	VELS, total		KiESEL, total			VELS		KiESEL		
Main food group	Mean	*N*	Mean	*N*^a^	Difference consumption	Mean	*N*	Mean	*N*^a^	Difference consumption
Grains and grain-based products	8.2	722	9.4	690 (703)	+13%*	4.0	85	5.7	47 (69)	+30%
Vegetables and vegetable products	2.5	623	3.4	579 (590)	+26%*	2.8	52	3.2	20 (37)	+11%
Starchy roots or tubers and products thereof	2.5	611	2.5	536 (540)	+2%	2.5	53	3.9	17 (32)	+34%
Legumes, nuts, oilseeds and spices	0.3	160	0.4	148 (142)	+26%	0.5	4	0.4	3 (5)	−16%
Fruit and fruit products	7.4	708	8.5	677 (687)	+13%	7.0	82	8.1	49 (67)	+13%
Meat and meat products	2.0	642	2.4	602 (608)	+20%*	1.3	41	1.5	21 (32)	+16%
Fish, seafood and invertebrates	1.0	246	1.2	234 (242)	+20%*	0.7	9	0.7	2 (6)	−2%
Milk and dairy products	17.9	688	13.8	660 (669)	−30%*	13.0	59	8.2	21 (40)	−59%
Eggs and egg products	0.8	278	1.1	214 (233)	+25%	0.5	8	0.8	1 (3)	+42%*
Sugar, confectionery and water-based sweet desserts	1.2	629	1.3	543 (549)	+6%	0.3	24	0.1	8 (6)	−144%
Animal and vegetable fats and oils	0.5	644	0.4	547 (580)	−3%	0.5	62	0.5	34 (46)	+9%
Fruit and vegetable juices and nectars	9.2	554	9.6	418 (411)	+4%	4.8	44	7.8	17 (18)	+39%
Water and water-based beverages	28.2	725	37.5	697 (710)	+25%*	45.4	95	62.4	57 (80)	+27%*
Coffee, cocoa, tea and infusions	7.6	494	7.6	368 (384)	−1%	10.2	45	9.7	9 (10)	−6%
Alcoholic beverages^b^	3.1	17	2.4	9 (8)	−29%	0.0	0	0.0	0	0%
Food products for infants and toddlers	19.1	296	14.2	282 (318)	−35%*	40.4	95	33.4	57 (80)	−21%*
Products for non-standard diets and food imitates	2.8	48	5.1	61 (57)	+45%*	5.5	5	0.7	3 (4)	−677%
Composite dishes	5.3	655	6.3	626 (640)	+15%*	5.9	46	9.2	35 (49)	+35%
Seasoning, sauces and condiments	1.2	552	1.4	527 (536)	+14%	1.0	20	0.9	7 (12)	−8%
TOTAL	93.7	732	99.2	701 (715)	+6%*	118.8	95	123.7	57 (80)	+5%

Age group	1–<3 y.					3–<5 y.				
Survey	VELS		KiESEL			VELS		KiESEL		
Main food group	Mean	*N*	Mean	*N*^a^	Difference consumption	Mean	*N*	Mean	*N*^a^	Difference consumption
Grains and grain-based products	8.6	340	10.3	306 (324)	+16%*	8.8	297	9.2	337 (310)	+4%
Vegetables and vegetable products	2.5	304	3.5	251 (271)	+28%*	2.4	267	3.3	308 (282)	+27%*
Starchy roots or tubers and products thereof	2.5	296	2.7	254 (257)	+6%	2.3	262	2.2	266 (251)	−5%
Legumes, nuts, oilseeds and spices	0.3	65	0.5	59 (59)	+45%	0.3	91	0.3	86 (78)	+6%
Fruit and fruit products	8.2	336	10.2	295 (313)	+19%*	6.5	290	7.1	333 (307)	+8%
Meat and meat products	2.0	318	2.4	268 (286)	+16%	2.0	283	2.5	313 (290)	+21%*
Fish, seafood and invertebrates	1.0	119	1.2	111 (115)	+13%	0.9	118	1.3	121 (121)	+26%*
Milk and dairy products	20.6	335	15.4	303 (320)	−34%*	15.7	294	12.6	335 (309)	−24%*
Eggs and egg products	0.8	131	1.1	97 (114)	+22%*	0.8	139	1.1	116 (116)	+26%*
Sugar, confectionery and water-based sweet desserts	1.1	315	1.1	224 (250)	−7%	1.4	290	1.5	312 (293)	+7%
Animal and vegetable fats and oils	0.5	305	0.5	252 (273)	+5%	0.5	277	0.4	261 (261)	−14%
Fruit and vegetable juices and nectars	9.9	267	9.7	169 (171)	−3%	9.2	243	9.7	232 (222)	+6%
Water and water-based beverages	29.0	335	39.6	306 (322)	+27%*	21.7	295	31.3	335 (308)	+31%*
Coffee, cocoa, tea and infusions	9.1	228	8.6	145 (165)	−5%	5.7	221	6.8	214 (209)	+17%
Alcoholic beverages^b^	3.9	5	5.9	2 (3)	+34%	2.7	12	1.6	8 (5)	−67%
Food products for infants and toddlers	10.3	161	10.3	164 (183)	0%	4.2	40	6.7	61 (55)	+38%
Products for non-standard diets and food imitates	2.6	27	5.2	35 (31)	+50%*	2.3	16	5.6	23 (22)	+58%*
Composite dishes	5.5	325	6.9	285 (302)	+21%*	5.1	284	5.4	307 (289)	+6%
Seasoning, sauces and condiments	1.2	277	1.4	229 (253)	+16%	1.2	255	1.4	290 (271)	+11%
TOTAL	99.3	340	106.6	308 (325)	+7%*	79.5	297	88.3	337 (310)	+10%*

*y.* year(s), *N* Number of consumers.

*Mean consumption value was significantly different, *p* < 0.05.

^a^weighted number is presented (unweighted sample size is given in parenthesis).

^b^group also includes malt beer.

### Approach 1

The existing MEAL food list 2016 covered 94% of the diet from the newly collected food consumption survey for children (KiESEL study) (Table 2). In addition, the MEAL food list 2016, based on food consumption data for young children and adults, also covered 91% of the diet for the middle age group of the EsKiMo II study. However, more detailed evaluations showed that the 90% criterion did not apply on main food group level and for different age groups. Especially the main food groups ‘Products for non-standard diets, food imitates’ and ‘Alcoholic beverages’^2^ were underrepresented. In line with this, ‘Products for non-standard diets, food imitates’ also showed significant changes in consumption, while ‘Alcoholic beverages’ showed a great relative decrease (non-significant) for the age group 3–<5 years (Table 1).

**Table 2:** Coverage of food consumption (%) for ‘Approach 1 food list’.

Survey	KiESEL			EsKiMo II	
Age group	0.5–<1 y.	1–<3 y.	3–<6 y.	6–<9 y.	9–<12 y.
Main food group					
Grains and grain-based products	91	90	91	90	90
Vegetables and vegetable products	**80**	96	97	97	97
Starchy roots or tubers and products thereof	99	97	97	96	97
Legumes, nuts, oilseeds and spices	100	90	**81**	**87**	**85**
Fruit and fruit products	**86**	96	95	96	96
Meat and meat products	90	**86**	**80**	**83**	**84**
Fish, seafood and invertebrates	100	**89**	91	**82**	94
Milk and dairy products	100	98	99	94	96
Eggs and egg products	100	100	100	99	100
Sugar, confectionery and water-based sweet desserts	100	**87**	**86**	**85**	**78**
Animal and vegetable fats and oils	**81**	**84**	**86**	90	91
Fruit and vegetable juices and nectars	**85**	94	96	98	96
Water and water-based beverages	100	95	92	**89**	**89**
Coffee, cocoa, tea and infusions	100	100	100	100	100
Alcoholic beverages	—	**63**	**70**	100	100
Food products for infants and toddlers	98	93	98	97	100
Products for non-standard diets and food imitates	**36**	**58**	**30**	**40**	**71**
Composite dishes	**85**	**89**	90	**89**	**86**
Seasoning, sauces and condiments	100	**84**	90	90	91
TOTAL	94			91	

Shown is the coverage per main food group and over the total food list. Numbers in bold emphasize coverage <90%.

### Approach 2

Approach 2 revealed, which foods should be added to comply with the 90% criterion also on age group or main food group level. Table 3 shows food consumption of the TDS diet based on the ‘Approach 2 food list’, the increase of coverage per main food group along with the impact on the MEAL food list, when additional MEAL foods are selected to reach coverage of  ≥90% food consumption. For KiESEL 21 and for EsKiMo II 16 additional foods were identified as being part of 90% of the diet. In total, 32 foods were added to the original food list 2016, increasing the list from 356 to 388 foods (Supplementary Material Table S2). Main food groups with highest increase in foods were ‘Legumes, nuts, oilseeds and spices‘ and ‘Composite dishes’ (*n* = 5 added foods) and’Products for non-standard diets’ as well as ‘Sugar, confectionery and water-based sweet desserts’ (*n* = 4 added foods). By the procedure applied, a coverage of 88–100% and 90–100% was achieved for KiESEL and EsKiMo II, respectively. Due to a high consumption proportion of not further specified nuts, the coverage of 90% could not be achieved for the main food group ‘Legumes, nuts, oilseeds and spices’ in the KiESEL study. This is also the reason for inclusion of five additional foods, since all consumed foods from this group needed to be selected for the age group 3–<6 years in order to increase coverage of food consumption to at least 88%. For all five foods, the consumer rate was <5%. For 23 of the 32 foods the proportion of consumers is <5%. Nine foods complied with both, being part of 90% of food consumption and having ≥5% consumers. If just foods with ≥ 5% consumers would be selected, then just these nine additional foods would be selected in total, but just a coverage between 30% (KiESEL) or 40% (EsKiMo II) to 100% would be achieved (Supplementary Material Table S3).

**Table 3:** Mean food consumption of TDS diet* per main food group (g/kg bw/day), changes in the number of foods (*n*) in the MEAL food list 2016 and coverage of food consumption (%) for ‘Approach 2 food list’.

	KiESEL				EsKiMo II			MEAL food list 2016	Approach 2 food list	Total	KiESEL, total	EsKiMo II, total
	0.5–<1 y.	1–<3 y.	3–<6 y.	change *n* MEAL foods	6–<9 y.	9–<12 y.	MEAL foods	*N* foods	*N* foods	change *n* MEAL foods	% coverage	% coverage
Main food group												
Grains and grain-based products	4.4	9.2	8.2	0	7.6	5.6	0	40	40	0	90–91	90
Vegetables and vegetable products	1.0	2.8	2.7	+2	2.5	1.5	0	34	36	+2	94–97	97
Starchy roots or tubers and products thereof	1.1	2.2	1.7	0	1.6	1.4	0	8	8	0	97 – 99	96–97
Legumes, nuts, oilseeds and spices	<0.1	0.1	0.1	+5	0.1	0.0	+2	20	25	+5	88^a^–100	93 –94
Fruit and fruit products	6.7	9.5	6.1	+1	4.6	2.7	+0	22	23	+1	96–97	96
Meat and meat products	0.5	2.0	2.2	+1	1.9	1.5	+1	35	36	+1	93–95	91–92
Fish, seafood and invertebrates	<0.1	0.4	0.4	+1	0.3	0.3	+2	30	33	+3	91–100	93–96
Milk and dairy products	3.1	14.8	11.2	0	8.2	5.7	0	23	23	0	98–99	94–96
Eggs and egg products	<0.1	0.3	0.3	0	0.3	0.2	0	2	2	0	100	99–100
Sugar, confectionery and water–based sweet desserts	<0.1	0.7	1.3	+1	1.1	0.8	+3	15	19	+4	94–100	90–95
Animal and vegetable fats and oils	0.3	0.4	0.3	+2	0.3	0.2	0	8	10	+2	97–100	99
Fruit and vegetable juices and nectars	2.4	5.0	5.3	+1	3.4	2.7	0	10	11	+1	99–100	95–97
Water and water-based beverages	62.2	37.4	28.4	0	27.5	23.1	+1	6	7	+1	92–100	93–94
Coffee, cocoa, tea and infusions	1.6	4.1	3.7	0	2.5	1.6	0	9	9	0	100	100
Alcoholic beverages	0.0	<0.1	<0.1	+1	<0.1	<0.1	0	8	9	+1	100	100
Food products for infants and toddlers	32.8	5.1	0.8	0	<0.1	<0.1	0	11	11	0	93–98	97–100
Products for non-standard diets and food imitates	<0.1	0.6	0.4	+2	<0.1	<0.1	+4	7	11	+4	96–100	94–98
Composite dishes	5.3	5.8	4.7	+2	4.4	3.7	+3	52	57	+5	91–93	90–91
Seasoning, sauces and condiments	0.1	0.9	1.1	+2	0.9	0.8	0	16	18	+2	91–100	90–91
TOTAL				+21			+16	356	388	+32	88–100	90–100

*y.* year(s) *TDS diet: consumption achieved by the intake of foods from the TDS food list. Shown is the average consumption over all individuals per age group.

^a^due to a high consumption amount of not further specified nuts 90% of coverage cannot be achieved.

### Approach 3

The ‘Approach 3 food list’ was compiled by reviewing food consumption amounts from KiESEL and EsKiMo II per age group and per main food group, selecting relevant foods to cover at least 90% of the diet and finally adding foods also relevant for adults (from NVS II study) from the MEAL food list 2016. Additionally, the screening for foods relevant for exposure lead to inclusion of two more foods. These were ‘Growing-up milk, >12 month (powder)’ and ‘Wholemeal pasta’. Latter was generated by disaggregating wholemeal products from the existing MEAL foods ‘Durum pasta’ and ‘Egg pasta’. Approach 3 resulted in a list of 391 foods (Supplementary Material Table S4). Greatest changes compared to the MEAL food list 2016 were again visible in the main food groups ‘Legumes, nuts, oilseeds and spices’, ‘Products for non-standard diets and food imitates’ and ‘Sugar, confectionery and water-based sweet desserts’ with a plus of six or five foods (Table 4). In the main food groups ‘Food products for infants and toddlers’ and ‘Milk and dairy products’ amount of foods was decreased by three or one foods (Table 4).

**Table 4:** Mean food consumption of TDS diet* per main food group (g/kg bw/day), changes in the number of foods (*n*) in the MEAL food list 2016 and coverage of food consumption (%) for ‘Approach 3 food list’.

	KiESEL			EsKiMo II		MEAL food list 2016	Approach 3 food list	Total	KiESEL, total	EsKiMo II, total
	0.5–<1 y.	1–<3 y.	3–<6 y.	6–<9 y.	9–<12 y.	*N* foods	*N* foods	change *n* MEAL foods	% coverage	% coverage
Main food group										
Grains and grain-based products	4.6	9.5	8.4	7.8	5.8	40	44	+4	93–95	93–94
Vegetables and vegetable products	1.0	2.8	2.7	2.5	1.5	34	35	+1	94–98	97–98
Starchy roots or tubers and products thereof	1.1	2.2	1.7	1.6	1.4	8	8	0	97–99	96–97
Legumes, nuts, oilseeds and spices	<0.1	0.1	0.1	0.1	<0.1	20	26	+6	88^a^–100	93–94
Fruit and fruit products	6.7	9.5	6.1	4.6	2.7	22	23	+1	96–97	96
Meat and meat products	0.5	2.0	2.3	2.0	1.5	35	38	+3	95–96	94
Fish, seafood and invertebrates	<0.1	0.4	0.4	0.4	0.3	30	34	+4	93–100	94–97
Milk and dairy products	3.1	14.8	11.2	8.2	5.7	23	22	−1	98–100	94–96
Eggs and egg products	<0.1	0.3	0.3	0.3	0.2	2	2	0	100	99–100
Sugar, confectionery and water-based sweet desserts	<0.1	0.7	1.3	1.1	0.8	15	20	+5	94–100	92–94
Animal and vegetable fats and oils	0.3	0.4	0.3	0.3	0.2	8	9	+1	95–98	99
Fruit and vegetable juices and nectars	2.4	5.0	5.3	3.4	2.7	10	11	+1	94–98	96–98
Water and water-based beverages	62.2	38.3	29.2	27.5	23.1	6	7	+1	95–100	93–94
Coffee, cocoa, tea and infusions	1.6	4.1	3.7	2.5	1.6	9	9	0	100	100
Alcoholic beverages	0.0	<0.1	<0.1	<0.1	0.1	8	9	+1	100	100
Food products for infants and toddlers	30.8	5.1	0.8	<0.1	<0.1	11	8	−3	91–94	97–100
Products for non-standard diets and food imitates	0.0	0.6	0.4	0.1	0.1	7	12	+5	96–100	98
Composite dishes	5.3	5.8	4.8	4.4	3.7	52	55	+3	91–93	90–91
Seasoning, sauces and condiments	0.1	0.9	1.1	0.9	0.8	16	19	+3	91–100	93
TOTAL						356	391	+35	88*–100	90–100

*y.* year(s) *TDS diet: consumption achieved by the intake of foods from the TDS food list. Shown is the average consumption over all individuals per age group.

^a^due to a high consumption amount of not further specified nuts 90% of coverage cannot be achieved.

**Fig. 2:**
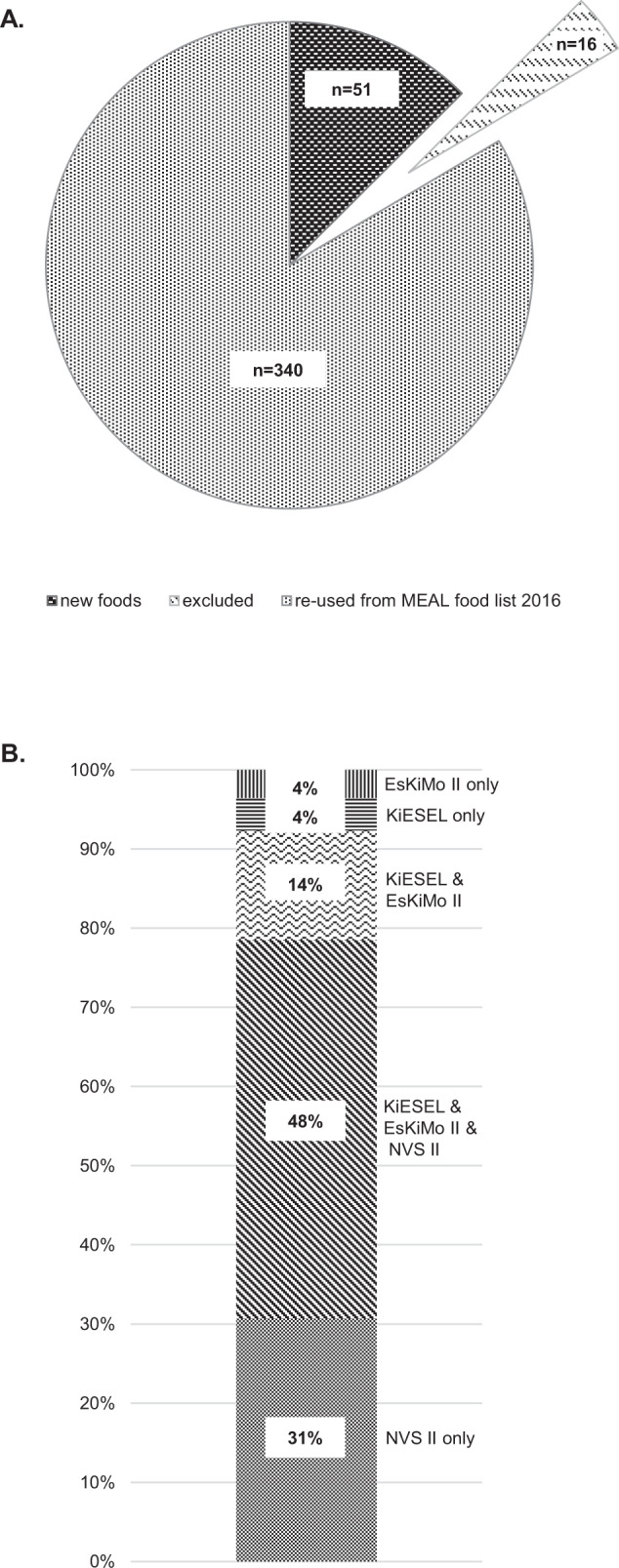
Composition of the ‘Approach 3 food list‘. **A** Comparison with MEAL food list 2016. **B** Composition according to the underlying food consumption surveys (age groups covered by the surveys: KiESEL: 0.5–<6 years; EsKiMo II: 6–<12 years; NVS II: 14–80 years).

Figure 2 visualizes the reusability of the MEAL food list 2016 if a complete new selection was adopted based on new food consumption data. 340 foods were similar to the food list 2016, which corresponded to 96% of the original 356 foods. 16 foods of the original food list were no longer relevant (Supplementary Material Table S5) and 51 foods were newly included (Fig. 2A). Figure 2B further differentiates the foods related to the origin of the survey by which they became relevant. 31% of the ‘Approach 3 food list’ was relevant for adults only (NVS II). About half of the food list was relevant for all considered age groups (48%). 21% were included because of relevance for KiESEL and/or EsKiMo II only.

Forty-six of the 391 foods have consumer rates <5% (Supplementary Material Table S4). Likewise in approach 2, adopting the ≥5% consumer criterion would decease coverage of the diet considerably (Supplementary Material Table S6). However, in contrast to approach 2, 20 instead of nine new foods were identified complying with being part of 90% of food consumption plus having ≥5% consumer rate.

## Discussion

The BfR MEAL Study is a comprehensive project mandated by the Federal Ministry of Food and Agriculture (BMEL) and funded with about 13 million euro. Data on approximately 300 substances in >350 foods were collected 2016–2021. The data complement the existing food monitoring activities in many ways and improve consumer safety in Germany [10]. The already existing data basis from the BfR MEAL Study and the established infrastructure provide a valuable environment for ongoing data updates. The presented evaluations focused on the food list of the BfR MEAL Study, and how the accuracy during its establishment in 2016 leads to reusability and comparability, i.e., whether or to which extent the original food list is still applicable for future data usage or collection. The two hypotheses, that (1) the detailed food list of the BfR MEAL Study is also covering ≥90% of the diet of updated food consumption data and (2) that only consideration of young children and adults is enough to also cover food consumption of middle age groups in Germany, were approved by approach 1. Ninety-four percent and 91% of food consumption of updated data for children (KiESEL) and for the middle age group (EsKiMo II) was covered by the original food list 2016. Hence, the initial goal to select about 350 foods for the TDS food list when planning the BfR MEAL Study turned out to be a reasonable number when compiling a TDS food list, not only in terms of capturing average food consumption of diverse population groups, but also in terms of reusability for future food consumption updates. This was also supported by findings from approach 3, which showed that 96% of the original food list would also be chosen according to the new food consumption data (with the restriction that this is just based on data for children and parts of the food list originate from the NVS II data for adults).

Selecting at least 90% of food consumption for a TDS is recommended by EFSA, FAO and WHO [1] in order to capture the population’s food consumption and thus the population’s exposure. Nevertheless, when applying this criterion to overall food consumption, coverage is mainly driven by high contributing food groups, such as beverages or grain products. Foods or food groups with lower consumption could partly or completely be neglected. Therefore, more detailed selection based on main food groups and different age groups is useful, especially when it comes to exposure assessment for population subgroups. This became visible, when elaborating the coverage of approach 1 in more detail. This more differentiated evaluation revealed underrepresentation of certain main food groups or age groups, when applying the MEAL food list 2016 to the new food consumption data (Table 2), which would result in an underestimation of the exposure for concerned subgroups.

Strategies to deal with <90% coverage were evaluated in approaches 2 and 3. Both approaches lead to ≥90% coverage per main food group and per age group, however with different effort and different outcome in number and type of selected foods. Approach 2 identified 32 and approach 3 identified 51 additional foods, which need to be added to achieve ≥90% coverage on the detailed level. The reason for the lower amount of foods in approach 2 was that additional foods were just included when 90% coverage of food consumption was not achieved by the original food list. Approach 3 instead aims at reaching ≥90% coverage by rebuilding a completely new food list. Therefore, more foods per age group are included. All but three foods, identified from approach 2 are also included in approach 3 food list. That shows that approach 2 captures a great proportion of additionally relevant foods, but also that approach 3 does better fit the specific food consumption patterns of the new surveys and thus will result in more accurate exposure assessments. Furthermore, approach 3 allows identification and removal of foods no more relevant based on new food consumption data. This is important, when setting up a new sampling plan and helps to save resources in food sampling and analysis. Nevertheless, already collected data from those foods should still be included in exposure assessment in order to use as much information as possible. Although approach 3 seems to be the most accurate update strategy, it hast to be kept in mind, that the more changes to the food list are made, the less comparable will repeated TDS studies be. Therefore, next to effort and resources also the potential standardization in repeated sampling should be a criterion when updating a food list. In both approaches, the number of foods increased from 356 MEAL foods to 388 or 391. A possible explanation could be that the observed change was rather driven by an increase in product variability on the market (e.g., various vegetarian products) than by a change in food choices. Nevertheless, it has to be considered, that selection of foods for a TDS food list is always a trade-off between scientific needs and available resources. In the here applied approaches 2 and 3 the criteria of being part of 90% of food consumption or having at least 5% of consumers was consequently applied per age group. This leads to a theoretical maximum food list. In some cases, it would be reasonable to exclude more foods or to reduce the number by aggregating some of the relevant foods into a common MEAL food. E.g. increasing the coverage of ‘Alcoholic beverages’ for KiESEL from 63 or 70% to >90% in approach 2 was achieved by including just one more food (‘White wine/sparkling wine, non-alcoholic’) with only four consumers (Tables 2 and  3). However, the impact on coverage was high since few individuals consume just a few foods in this group. In those cases, removing those foods would be acceptable. In other cases, such as ‘Products for non-standard diets and food imitates’ the different foods included were very variable with few consumers for each. In total, four foods need to be added to achieve a >90% coverage. Excluding those foods would reduce coverage down to 30% (Tables 2, 3 and Table S3). In such cases, further research of food consumption habits for specific populations groups or diets, or the inclusion of substance specific experts and experts of exposure and risk assessment could help to further narrow the food list. Especially the latter is a crucial step, since not only consumption and its coverage decide about exposure, but also the occurrence and variability of the substances under investigation. Low consumed foods can have great impact on exposure if they potentially contain high levels of contaminants. Furthermore, high concentration foods should not be aggregated with foods of lower concentrations in order to identify and separately analyse exposure associated to these foods. This aspect has not yet been considered in the present evaluation and must be taken into consideration after selecting chemicals for a follow-on TDS.

Just few publications report on updated TDS food lists related to new food consumption data. Egan et al. (2007) [6] updated the US FDA’s TDS food list from 1990 in 2003 based on new food consumption data for children and adults. Foods were grouped based on the similarity of their major ingredients. The most representative foods in terms of quantity were selected for the food list. This procedure corresponds to approach 3 of this study. The number of TDS foods remained similar with about 290 items in both US food lists. 75% of foods were common to both versions. Of the remaining 25% some were modified (e.g., other fat content) and about 15% were newly included. Although food consumption habits evolve differently in different countries and the present study just considers changes in child food consumption, the observed proportion of change is in line with 13% newly included foods in approach 3 in the present study. Sirot et al. (2009) [11] report on the update of the 1999 food list from the first French TDS to the second French TDS in 2006. The food list of the first French TDS 1999 contained 338 foods and was compiled according the approach of the US FDA’s TDS food list [11, 12]. The second French TDS used updated consumption data but adopted another approach for food selection. A predefined number of foods was selected according to the criteria of representing most consumed foods in terms of quantity (not necessarily covering 90% of the diet) plus having at least 5% of consumer rate. In addition, known high contributors to exposure with regard to contaminants of interest were added. In total, 186 foods were finally included covering 82–91% of the diet [11]. This procedure is almost comparable to approach 3 when just foods with ≥5% consumers are included. In the present study, this approach would overall (i.e., measured over the total diet) cover approximately 93%. If foods relevant for adults only (NVS II) would be excluded from this calculation, coverage would be 89–91% (data not shown). Devlin et al. (2014) [13] compared different approaches to compile TDS food lists based on food consumption data from 14 countries from the EFSA Comprehensive Food Consumption Database. The approaches comprised (1) selection of foods covering >90% of diet based on main food group level, (2) excluding foods <5% consumer rate from the first approach, (3) selection of foods covering >90% of the diet based on the overall diet, and (4) excluding foods <5% consumer rate from the third approach. According to their results, all approaches reached at least 85% coverage of the diet. The authors also showed that exclusion of <5% consumer foods had small effect on overall coverage (about 2%), whereas the number of selected foods was considerably decreased by that approach for some countries. The here presented results confirm that overall food consumption was also covered by about 89 to 91% when excluding foods with <5% consumer rate. Indeed, it can be argued that this is acceptable given the fact that many foods can be excluded – and thus many resources saved for shopping, processing and analysis of these foods – while just losing about 2% [13] of overall coverage. However, when it comes to subgroup level (main food group or population subgroups) and applying 5% consumer rate cut-off, coverage was decreased down to 30% (Table S3) or even 2% (Table S6) per main food group. Hence, it turns out, that resources can be saved by considering the exclusion of foods with low consumer rate. Nevertheless, this needs to been seen in the context of subgroups and should to be decided case-by-case on expert based judgments to achieve a targeted and resource saving strategy.

## Conclusion

The presented assessments proof reusability of the MEAL food list 2016 in different ways. The approach chosen when establishing the MEAL food list in 2016 has turned out to be comprehensive enough to cover also 90% of the consumption of newly collected food consumption data for children in Germany. In addition, the resource-saving approach to just consider young children and adults was sufficient to also cover indirectly the food consumption habits of the middle groups and standardization of food lists seems to be feasible without violating the 90% criteria for TDS.

Approach 1 turned out to be a quick and effective strategy, when new food consumption data become available. It has the least effort, requires the least resources, and in addition it has the advantage that there are no changes to the original food list, which makes results comparable for repeated TDS samplings. Nevertheless, in cases where comparability of food lists is not the priority, more accurate selection strategies could be applied to avoid underrepresentation and thus underestimation of exposure of certain main food groups or subpopulations. Approach 2 and 3 mainly differ in the effort. Thus, their application depends on available resources for evaluation of new food consumption data, and sampling and analysis of new foods. Both approaches lead to coverage of ≥ 90% of food consumption in different age and food groups. Approach 2 just added new foods to the existing list. Therefore, the coverage of an already existing food list can quickly be adapted. Approach 3 identified more accurately new foods and allowed exclusion of no more relevant foods. This allows more accurate exposure assessments for population sub-groups and helps to safe resources in case of repeated TDS sampling. However, when following trends, the changes in the food list need to be carefully evaluated, when comparing repeated measurements.

If and how low consumer rate foods should be excluded should be a case-related decision.

In conclusion, the great effort put into the initial food MEAL food list 2016 now allows a quick and resource saving update for future TDS activities in Germany.

### Supplementary information

Reporting Checklist

Supplementary Material

## Data Availability

Food consumption data for children (0.5–<5 years) from the VELS study [5] is available in the EFSA Comprehensive European Food Consumption Database (https://www.efsa.europa.eu/en/data-report/food-consumption-data). Furthermore, data publishing via a data repository linked to the homepage of the currently ongoing FNS-Cloud project (Food Nutrition Security Cloud; FNS-Cloud) (https://www.fns-cloud.eu/) is in progress. A scientific use file for the KiESEL food consumption data for children (0.5–<6 years) [7] is in preparation and will be made available via the BfR webpage https://www.kiesel-studie.de. Food consumption data from the EsKiMo II study for children (6–<12 years) years [8] were provided by Robert Koch Institute (RKI), Department of Epidemiology and Health Monitoring, within the framework of data provision for exposure and risk assessment at the German Federal Institute for Risk Assessment (BfR) and are not publicly available at the point of preparing this study. Information about public use are available at https://www.kiggs-studie.de/ergebnisse/kiggs-welle-2/scientific-use-file.html.

## Footnotes

1 Main food group ‘Major isolated ingredients, additives, flavors, baking and processing aids’ was not considered since these foods are included in foods-as-eaten.

2 Alcoholic beverages also include beverages such as malt beer or non-alcoholic wine
